# Interdisciplinary Approach to a Tooth with Open Apex and Persistent Sinus

**DOI:** 10.1155/2015/907324

**Published:** 2015-09-06

**Authors:** Anoop N. Das, Krishnamohan Geetha, Ajay Varghese Kurian, Radhakrishnan Nair, K. Nandakumar

**Affiliations:** ^1^Department of Conservative and Endodontics, Azeezia College of Dental Sciences and Research, Meeyannoor, Kollam, Kerala 691537, India; ^2^Department of Periodontics and Implantology, Azeezia College of Dental Sciences and Research, Meeyannoor, Kollam, Kerala 691537, India

## Abstract

Traumatic injuries in childhood may disrupt root development leading to a tooth with open apex. Apexification procedures in such cases aim at root end closure after reasonable period of time. In some chronic cases, complete healing of the periapical area does not occur resulting in development of a nonhealing sinus. Failure of nonsurgical approach in such cases needs surgical intervention permitting thorough periapical curettage. In the present case, apexification procedure with MTA achieved root end closure but failed to heal the sinus for which surgical treatment was completed with thorough periapical curettage and application of platelet rich fibrin (PRF) and a combination of *β*-tricalcium phosphate and hydroxyapatite resulted in healing.

## 1. Introduction

Trauma or infection to a tooth at an early age causes interruption in root development leading to incomplete root formation with a wide funnel shaped canal [1]. Young people are more prone to injuries at the time when the root development is incomplete. These injuries are associated with a significant risk of teeth losing their vitality. The maxillary central incisor is the most commonly affected tooth in both dentitions. Lack of root development can provide a challenging clinical situation since cleaning, shaping, and obturation during root canal therapy may become difficult and unpredictable [2]. The traditional approach to the treatment of nonvital teeth with incompletely developed roots has been apexification using calcium hydroxide. The barrier formed facilitates the placement of an appropriate root-filling with a seal within the root canal. The introduction of mineral trioxide aggregate (MTA) led the way to a more definitive treatment and prognosis of apexification procedures.

Nonsurgical approach may fail to achieve complete healing because of persistent infection. Surgical intervention is required in such cases to treat a nonhealing sinus. The beta-tricalcium phosphate (*β*-TCP) and hydroxyapatite (HA) are alloplast widely used in periapical surgery to enhance new bone formation. These are osteoconductive bone grafts which get chemically resorbed with a concomitant release of bioactive ions [3]. The advantage of using the combined graft *β*-TCP and hydroxyapatite (Sybograf-T, Eucare Pharmaceuticals Ltd., India) is that *β*-TCP resorbs much faster, and the presence of hydroxyapatite in the structure reduces the resorbability of the material [4]. Also it allows better control of the bioabsorbable ability of the graft material resulting in accelerated new bone formation [5]. Platelet rich fibrin (PRF) is a second-generation platelet concentrate enriched with platelets and growth factors which promote periapical tissue regeneration and healing. It has been successfully used with bone grafts like *β*-TCP and hydroxyapatite for bone regeneration in the treatment of periodontal defects [6].

The present case describes an interdisciplinary approach to a tooth with open apex and a draining sinus. Apexification procedure with MTA failed to heal the sinus for which surgical treatment was completed with the application of platelet rich fibrin (PRF) and a combination of *β*-tricalcium phosphate and hydroxyapatite.

## 2. Case Report

**Figure 1:**
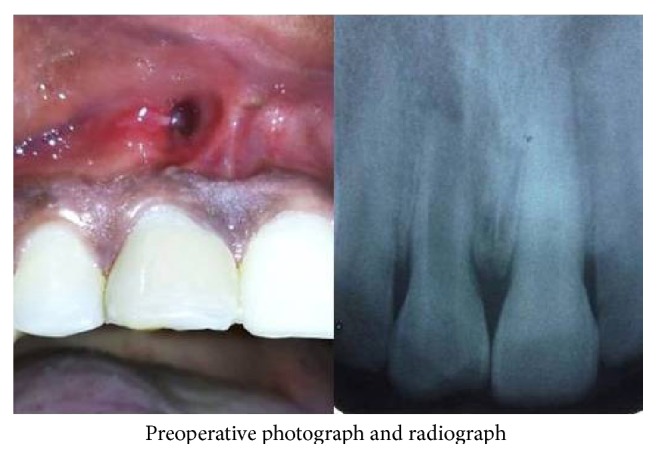
Preoperative photo- and radiograph.

**Figure 2:**
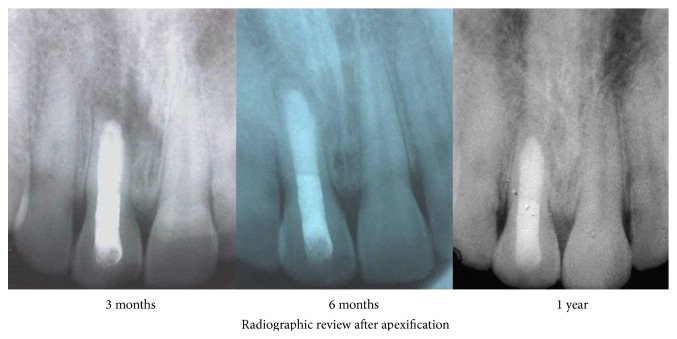
Postoperative radiographs after apexification.

A twenty-year-old male patient reported to the department with the chief complaint of tooth discoloration and constant pus discharge in relation to upper right central incisor (Figure 1). Past dental history revealed trauma of twelve years' duration and had not undergone any treatment for the affected tooth. Intraoral examination showed a pus discharging sinus in relation to upper right central incisor and radiographic examination revealed open apex. It was a case of chronic periapical abscess of right upper central incisor with an open apex. It was decided to do a nonsurgical approach to close the apex as the root tip appeared thin and fragile. The root canal was accessed and thoroughly irrigated with 3% sodium hypochlorite solution and saline as final rinse. Biomechanical preparation of the root canal was done using standard hand instruments (K-files) up to size #90. One-visit MTA apexification procedure was chosen as it was more predictable and less time consuming. Mineral trioxide aggregate (Angelus, Brazil) was mixed and slowly packed into the canal till the apical area was densely filled. A moist cotton pellet was placed over the MTA and the patient was recalled after one week and rest of the canal was obturated with gutta-percha. Radiograph was taken and evaluated every 3 months. Tooth was asymptomatic and one-year follow-up showed radiological signs of healing (Figure 2). But the periapical sinus did not heal even after a year.

**Figure 3:**
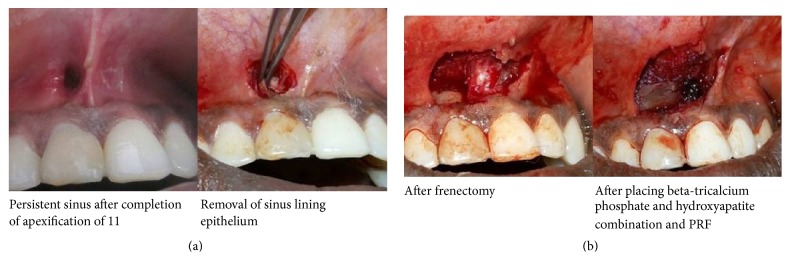
Sinus removal and graft placement.

**Figure 4:**
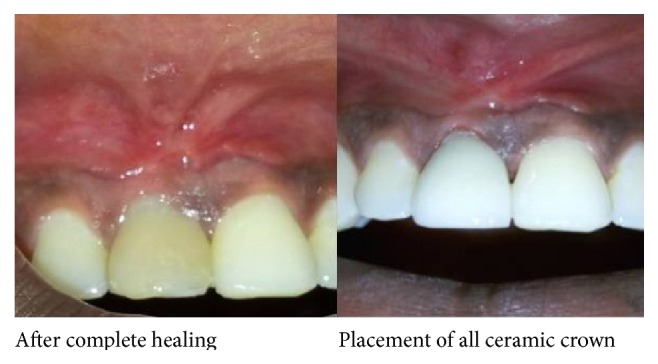
Postoperative photograph with all ceramic crown.

Surgical option was chosen to treat the nonhealing sinus. The lining epithelium of the sinus was excised and removed using tissue forceps and scissors. The frenal attachment adjacent to the sinus opening was also removed to avoid the tissue tension. Periapical curettage was done through the surgical opening (Figure 3). A combination of beta-tricalcium phosphate and hydroxyapatite (Sybograf-T) was placed at the surgical site. Platelet rich fibrin was prepared in accordance with the protocol developed by Freymiller and Aghaloo. Intravenous blood (by venipuncturing of the antecubital vein) was collected in a 10 mL sterile tube without anticoagulant and immediately centrifuged at 3,000 rpm for 10 minutes. PRF was separated from red corpuscles base using sterile tweezers just after removal of PPP (platelet-poor plasma) and then transferred into a sterile dappen dish and was placed over the site. The tissues were approximated and sutured using resorbable Vicryl 3-0 black suture. The patient was back after one week for suture removal and the wound healing was found to be satisfactory. The complete healing was achieved at 4-week time. Discoloration of the tooth was managed by all ceramic crown and patient was recalled for further review (Figure 4).

## 3. Discussion

Periapical infection of a tooth with chronic abscess, if left untreated, often forms a sinus tract, thereby allowing the pus to drain to the surface. Normal healing of such cases is unpredictable due to the complex nature of highly resistant endodontic microorganisms and fungi present in the periapical area, even after the source of infection is removed. Initiation of apical healing, regardless of the material used, takes at least 3-4 months and requires multiple appointments. Patient compliance with this treatment plan may be poor and many fail to return for scheduled visits. The temporary seal can also fail resulting in reinfection and prolongation or failure of treatment. The importance of the coronal seal in preventing endodontic failure is well documented [7–9]. For these reasons one-visit apexification has been suggested. Morse et al. defined one-visit apexification as the nonsurgical condensation of a biocompatible material into the apical end of the root canal. The purpose is to establish an apical stop that would enable the root canal to be filled immediately [10].

The use of triple antibiotic paste as an intracanal medicament has been reviewed widely by Trope [11]. Triple antibiotic paste contains both bactericidal (metronidazole and ciprofloxacin) and bacteriostatic (minocycline) agents to allow for successful revascularization. Some variations on the original triantibiotic paste mixture have been used with good success [12]. The concern of the antibiotic paste is that it may cause bacterial resistance. Additionally, minocycline may cause tooth discoloration. Development of resistant bacterial strains, tooth discoloration, and need of multiple visits can be considered as drawbacks of this technique [13], and therefore it was not chosen for the present case.

Calcium hydroxide has been the material of choice for apexification and has been widely reviewed and reported. MTA could be considered as an alternative to calcium hydroxide for apexification of permanent immature teeth. MTA consistently showed less inflammation, hyperemia, and necrosis, with more odontoblastic layer formation, than with calcium hydroxide [14]. This material is hydrophilic powder that sets in the presence of moisture [15]. It has superior biocompatibility and it is less cytotoxic due to its alkaline pH. The presence of calcium and phosphate ions in its formulation results in an ability to attract blastic cells and promote favorable environment for cementum deposition. A number of authors have reported clinical success using MTA for one-visit apexification [16, 17]. Sarris et al. used MTA as an apical plug in 17 incisors; of these 94.1% were assessed as being successful clinically, whereas 76.5% were reported to be successful radiographically. Current data show that MTA can be used as an apical barrier in teeth with necrotic pulp and open apex. More investigations are needed to prove its long term efficacy [18].

Presence of nonhealing sinus even after apexification necessitated surgical intervention. It may be due to the presence of epithelial lining and highly resistant microorganisms. In the present case the defects were filled with a combination of PRF. The ideal graft material must be osteoconductive and bioabsorbable to allow for host bone growth. So, beta-tricalcium phosphate and hydroxyapatite alloplast may be suitable as a bone fill material, since it potentially possesses both osteoconductive bone forming properties and the ability to be bioabsorbed. PRF is easy to prepare and the production time is shorter. The advantage of the second-generation thrombocyte concentration PRF to the first-generation thrombocyte concentration platelete rich plasma is biological activation [19, 20]. The combination with *β*-tricalcium phosphate and hydroxyapatite provides the advantages like PRF activation on the protein structure in the autogenous grafts and osteoblasts tended to adhere that were called into the environment area. PRF accelerates the healing effect by keeping the particles of graft together via its adhesive property. Del Fabbro et al. concluded from their study that the combined use of PRF with graft materials in bone defects contributes to wound healing [21]. Different studies have shown that there is complete biodegradation and new bone formation when *β*-TCP and hydroxyapatite are used in bone defect [22]. After obtaining complete sinus closure, a ceramic crown was fabricated which managed the aesthetic part.

## 4. Conclusion

Single visit apexification with a biocompatible material like MTA is effective in management of teeth with open apex. This procedure is predictable and less time consuming. Moreover, MTA with its regenerative potential has superior sealing properties and its moisture tolerance appears to offer the predictable option available till date. Persistent nonhealing sinus always proves to be an endodontic challenge. Periapical curettage and placing an innovative combination of PRF with *β*-tricalcium phosphate and hydroxyapatite proved successful for complete sinus closure and healing. The authors conclude that the present case proved to be successful by this interdisciplinary approach with reference to predictability, patient compliance, and time for the completion of treatment.
